# *Lichtheimia blakesleeana* as a New Potencial Producer of Phytase and Xylanase

**DOI:** 10.3390/molecules16064807

**Published:** 2011-06-09

**Authors:** Maria Luiza Carvalho Neves, Milena Fernandes da Silva, Cristina Maria Souza-Motta, Michele Rigon Spier, Carlos Ricardo Soccol, Tatiana Souza Porto, Keila Aparecida Moreira, Ana Lúcia Figueiredo Porto

**Affiliations:** 1 Renorbio/ UECE, Av. Paranjana, 1.700, 60740-000, Fortaleza, Ceará, Brazil; 2 Department of Pathology, University of Pernambuco, ICB-UPE, Rua Arnóbio Marques, 310, 50100-130, Recife, Pernambuco, Brazil; 3 Department of Morphology and Animal Physiology, Federal Rural University of Pernambuco, Av. Dom Manoel de Medeiros, s/n., 52171-900, Recife, Pernambuco, Brazil; 4 Department of Mycology, Federal University of Pernambuco, Av. Prof. Moraes Rego, 1235, 50670-901 Recife, Pernambuco, Brazil; 5 Department of Biotechnology and Bioprocess, Federal University of Paraná, P.O. Box 19011, 81531-970, Curitiba, Paraná, Brazil; 6 Academic Unit of Garanhuns, Federal Rural University of Pernambuco, Avenida Bom Pastor, s/n, Boa Vista, 55292-270, Garanhuns, Pernambuco, Brazil

**Keywords:** phytase, xylanase, citrus pulp, solid-state fermentation, *Lichtheimia blakesleeana*

## Abstract

Brazil is known for its great potential for production of renewable resources such as agro-industrial residues. These residues can be used as alternative sources of new products. Meanwhile, solid-state fermentation, with its advantages of energy conservation and pollution reduction, has been identified as a process of great potential for the production of bioactive compounds, especially enzymes. In the present work, a 2^3^ factorial design was used to evaluate the effects of pH, temperature and moisture on the production of phytase and xylanase by *Lichtheimia blakesleeana* URM 5604 through the fermentation of citrus pulp. Statistical analyses of the results showed that the only the pH influenced the production of these enzymes, with the best phytase production (264.68 U/g) ocurring at pH 6.0, 34 °C, initial moisture 50%, after 48 hours of culture. The best conditions for xylanase production (397.82 U/g) were fermentation for 120 hours at pH 4.0, 26 °C and initial moisture of 70%. The best parameters for the simultaneous production of phytase (226.92 U/g) and xylanase (215.59 U/g) were determined to be initial moisture of 50%, pH 6.0, 26 °C, and 48 hours of fermentation.

## 1. Introduction

Large numbers of microorganisms, including bacteria, yeasts and fungi, have been used in solid state fermentation (SSF) systems [1]. The use filamentous fungi for the production of commercially important products through in SSF has attracted much research interest during recent years [2,3]. Various fungi such as *Thermomucor* spp., *Mucor* spp., *Aspergillus* spp. and *Rhizopus* spp. have been used to produce various enzymes (cellulases, xylanases, ligninases, and pectinases), proteins and organic acids that can increase the digestibility of feed and access to plant nutrients [4,5,6]. 

Phytases are enzymes that catalyse the dephosphorylation of phytate in a stepwise manner to degrade inositol phosphate esters (*i.e.*, myo-inositol pentaphosphate to myo-inositol monophosphate) [5,7]. Phytase makes the phosphorus (P) from phytin available for animal digestion [8]. Reduction or elimination of inorganic phosphate supplementation of animal feed reduces P the levels in manure by about 33%, thus cutting the pollution burden by one-third [9]. Up to 80% of the total P content in plants may be present in the form of phytate and is thus effectively unavailable for monogastric or agastric aquatic animals [10].

Another enzyme found in cereals, xylanase, can be used in the diet of monogastric animals to hydrolyse non-starch polysaccharides such as β-glucans and arabinoxylans. This enzyme, also called endo-β-1,4-xylanase (EC 3.2.1.8), hydrolyses xylan, which is the major polysaccharide that makes up hemicelluloses. The presence of high levels of polysaccharides in animal diet results in lower feed conversion and slower weight gain [11,12].

The current trend in industries to use solid-state fermentation for the production of enzymes of fungal origin is due to the simplicity of the culture. The substrate can be used directly or enriched with nutrients, and the products of interest can be obtained in their concentrated form, thus facilitating downstream purification [3,9,13]. Citrus is the most abundant crop in the World [14] and Brazil is the second largest producer [15]. Oranges, lemons, grapefruits and mandarins represent approximately 98% of the entire industrialized crop; oranges, accounting for approximately 82% of the total, are the most relevant [16]. During the processing of oranges, a residue known as citrus pulp (CP) [15] composed of 60–65% peel, 30–35% segment pulp and 0–10% seeds is produced. Citrus seeds have a high percentage of protein, ether extractables and crude fibre [17]. They are equivalent to approx. 50% of the processed fruit mass and are part of citrus pulp [18]. The objective of this study was to investigate the effects of pH, temperature and initial moisture on the production of phytases and xylanases by *Lichtheimia blakesleeana* URM5604 using citrus pulp as substrate for solid state fermentation.

## 2. Results and Discussion

The *Lichtheimia blakesleeana* URM5604 used in this work has not been previously described as a producer of phytases and/or xylanases. We evaluated the enzyme production using solid-state fermentation at different pH, temperature and initial moisture values with citrus pulp as a substrate. This substrate has a low concentration of inorganic phosphate, which contributes to the production of phytase, as a high level of inorganic phosphate is known to repress phytase synthesis by filamentous fungi [15]. 

### 2.1. Optimization of Process Parameters for Phytase Production

Phytase activity values obtained from the 2^3^ tests with different fermentation time were presented in Table 1. With initial moisture 50%, pH 6.0, 34 °C and 48 hours of fermentation (run 7), *Lichtheimia blakesleeana* achieved the most efficient phytase production (264.68 U/g). 

**Table 1:** Phytase activity (U/g) in SSF during 120 hours of fermentation by *Lichtheimia blakesleeana* URM5604 using citrus pulp as substrate.

Runs	Time (hours)				
	24	48	72	96	120
1	98.16	123.48	161.77	126.74	130.76
2	67.36	95.04	118.43	121.70	74.24
3	150.88	226.92	198.82	195.48	129.79
4	125.28	154.48	142.80	112.71	106.84
5	120.23	173.81	226.05	103.08	101.65
6	159.83	122.18	175.43	69.78	72.01
7	188.50	264.68	131.82	132.00	128.07
8	204.29	242.80	100.31	93.49	101.03
9	205.22	157.65	178.79	95.57	88.45
10	180.12	143.02	93.50	72.22	107.03
11	172.16	157.07	101.86	87.15	113.67
12	162.98	198.46	94.74	99.96	95.39

**Figure 1:**
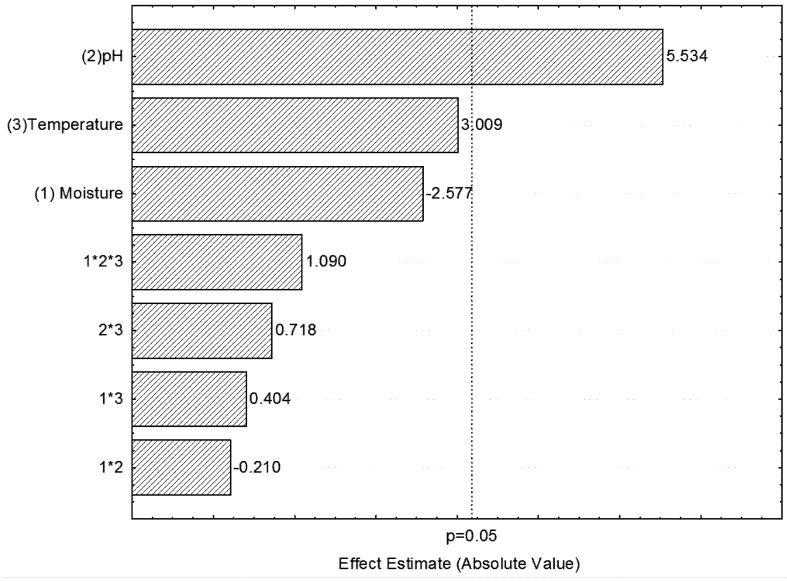
Pareto chart of main effects showing the production of Phytase during the 48 hours of fermentation of citrus pulp using three variables at two levels. The numbers in the ordinate of the figure refer to: (1) Moisture - percentage of water content, (2) pH and (3) Temperature.

The Pareto chart (Figure 1) shows the effects of pH, temperature and moisture, as well as their interactions with each other. Of all the variables studied, only pH was statistically significant for phytase production (p ≤ 0.05), and the maximum production of the enzyme was obtained when the highest pH value (6.0) was used. The other parameters such as temperature, moisture and their interactions did not influence phytase production. Moisture is one of the factors that interferes the most with the production of enzymes in solid-state fermentations. The addition of water causes swelling of the substrates and facilitates their use by microorganisms. However, with excessive moisture content there is a decrease in the transfer of O_2_ and the production of the enzymes [19]. On the other hand, low moisture reduces the diffusion of nutrients in the solid substrate, therefore reducing the moisture content and increasing the water-tightness of the substrate [20].

Similar results were reported by Chadha *et al.* [21] with *Rhizomucor pusillus*. The best phytase production (9.18 U/g substrate) was observed with wheat bran as substrate and initial moisture of 52%. However, Vassilev *et al.* [22] demonstrated better production of phytase (36.2 U/g DS) by *Aspergillus niger* NB2 at 70% moisture using olive waste (dry olive wastes) as substrate. El-Batal and Karem [19] used *Aspergillus niger* A-98 and showed better production of phytase with initial moisture of 60% when rapeseed meal was used as substrate. The water content of the substrate shows an important role in cell growth and enzyme production in solid-state fermentation, and the optimum amount of water varies with the substrate in each different system and the microorganism used [19]. 

### 2.2. Optimization of Process Parameters for Xylanase Production

Table 2 shows the xylanase activity values at different fermentation times, where maximum production of xylanase (397.8 U/g) by Lichtheimia *blakesleeana* was obtained with the highest water content (70%), pH 4.0, and 26 °C with 120 hours of fermentation (run 2). In this study, variables such as temperature and initial moisture content did not influence the production of xylanase. 

**Figure 2:**
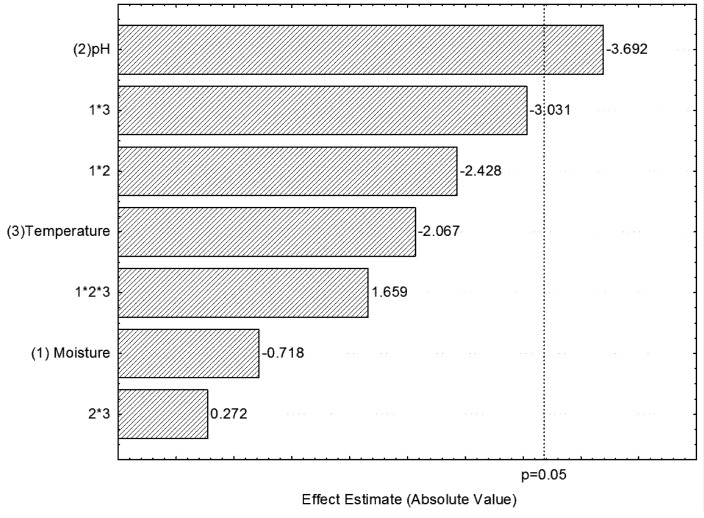
Pareto chart of main effects showing the production of xylanase during the 96 hours of fermentation of citrus pulp using three variables at two levels. The numbers in the ordinate of the figure refer to: (1) Moisture - percentage of water content, (2) pH and (3) Temperature.

As shown in Figure 2, the Pareto chart shows that the only significant factor (p ≤ 0.05) was pH, which negatively correlated with xylanase production, *i.e.*, the highest enzyme activity was achieved when the lowest pH was used (4.0). Botella *et al.* [20] demonstrated the best xylanase production by *Aspergillus awamori* with water content below 65% and using grape bagasse as substrate, which does not correlate with our results. The low enzyme activity was justified by the high levels of substrate moisture and the subsequent decrease of its porosity, changes in the structure of the particle, pasty texture, low oxygen transfer, as well as increase in the formation of aerial hyphae. Meanwhile, low moisture can reduce the diffusion of nutrients in solid substrates, thus decrease the degree of hydration and increase the tension of water. 

**Table 2:** Xylanase activity (U/g) in SSF during 120 hours of fermentation by *Lichtheimia blakesleeana* URM5604 using citrus pulp as substrate.

Runs	Time (hours)				
	24	48	72	96	120
1	174.93	198.53	222.17	222.28	291.70
2	158.27	165.89	164.46	384.25	397.82
3	221.31	215.59	229.23	225.38	214.57
4	173.76	190.85	189.38	180.49	177.24
5	48.51	85.25	174.67	281.76	288.51
6	31.11	74.92	107.29	206.35	273.62
7	62.05	142.44	180.60	214.69	185.30
8	118.05	195.73	140.22	100.34	96.50
9	58.54	95.80	155.65	176.45	226.69
10	87.95	115.90	136.53	205.05	195.85
11	56.96	110.75	130.11	229.88	261.11
12	63.43	127.71	134.19	147.08	179.72

Gervais and Molin [23] used *Thicoderma longibrachiatum* and *Aspergillus terreus* to produce xylanase and obtained optimum moistures of 55 and 75%, respectively. These results agreed with ours for *Lichtheimia blakesleeana* URM 5604, which produced 215.59 U/g xylanase with initial moisture of 50%, pH 6.0 and 48 hours of fermentation at 26 °C. 

The analyses of the effect variables in the 2^3^ full factorial design for both enzymes demonstrated that only pH showed a statistically significant effect, which is in negative correlation with xylanase production and in positive correlation with phytase production (Figure 1 and Figure 2). The interactions between the variables showed no statistically significant effects. 

### 2.3. The Best Conditions for Phytase and Xylanase Production

The optimum condition for the production of phytase were identified as initial moisture of 50%, pH 6.0, temperature 34 °C and 48 hours of fermentation. Under these conditions, *Lichtheimia blakesleeana* produced 264.68 U/g phytase (run 7, Table 1). The lowest production (67.36 U/g) occurred with 70% moisture, pH 4.0, and 26 °C fermentation for 24 hours (run 2). Singh and Satyanayana [1] showed that *Sporotrichum thermoplile* with sesame cake as substrate produced 348.76 U/g phytase with 120 hours of fermentation. In contrast, Chadha *et al.* [21] only obtained 9.18 U/g phytase by *Rhizomucor pusillus* using wheat bran with pH 8.0 and 48 hours of fermentation, which was 10 times lower than the lowest phytase production (95.04 U/g) obtained by us, with Lichtheimia *blakesleeana* and 48 hours of fermentation (run 2, Table 1). 

**Figure 3:**
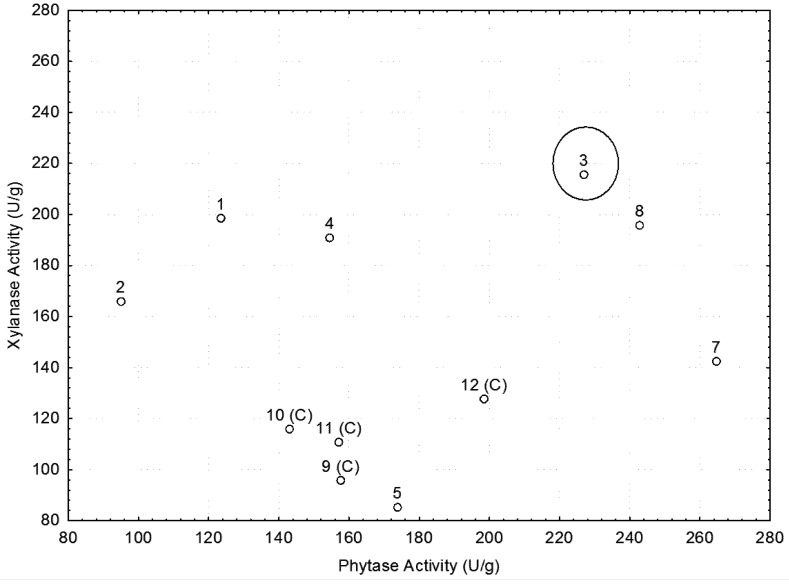
Simultaneous production of phytase and xylanase enzymes by *Lichtheimia blakesleeana* with initial moisture of 70%, pH 6.0, and 48 hours of fermentation at 26 °C.

The best conditions for xylanase production (397.82 U/g) occurred with initial moisture of 70%, pH 4.0, and 120 hours fermentation at a temperature of 26 °C, while the lowest production (31.1 U/g) occurred in run 6 (Table 2). Shah and Madamwar [24], working with *Aspergillus foetidus*, observed that initial pH significantly affected the production of xylanase, with the highest production of this enzyme (80.5 U/mL) being achieved at acidic pH (5.0) with 96 hours of fermentation. This finding was in agreement with ours and clearly demonstrated the acidophilic nature of the fungus because the fungal growth and the xylanase production nearly ceased at pH 8.0 and 9.0. However, our results are in disagreement with those obtained by Camassola and Dillon [25], who studied the production of xylanase by *Penicillium echinulatum* grown on sugar cane bagasse and wheat bran and observed that there was no direct relationship between increased pH and the production of xylanase. In contrast, our study showed that a proper choice of variables could enhance the production of these enzymes. The best condition for the simultaneous production of phytase enzymes (226.92 U/g) and xylanase (215.59 U/g) was initial moisture content of 50%, pH 6.0 and 26 °C incubation (Figure 3).

## 3. Experimental

### 3.1. Microorganism and Inoculum Preparation

*Lichtheimia blakesleeana* URM5604 was obtained from the URM Culture Collection of the Department of Mycology, Federal University of Pernambuco. The culture was grown and maintained on potato-dextrose-agar (PDA) slants. The slants were stored at 4 °C and sub-cultured fortnightly. Seven-day-old fully sporulated slant was used for inoculum preparation. For this, 2 mL sterile distilled water containing 0.1% Tween 80 was added to the slant and spores were scraped with a sterile needle. The number of spores was determined in a Neubauer counting chamber and the inoculum of 10^7^ spores/g CP (citrus pulp) was used for SSF.

### 3.2. Substrate

The CP was kindly supplied by the Department of Biotechnology and Bioprocess, Federal University of Parana, Brazil. This substrate was initially crushed in industrial mill with a final particle size of 0.8 and 2.0 mm. The moisture of the substrate was determined in accordance with the standards of the Institute Adolfo Lutz [26].

### 3.3. Experimental Design of Solid-State Fermentation

Fermentations were carried out using 250 mL Erlenmeyer flasks. Ten grams of residue (8.0 grams of citrus pulp with a diameter of 2.0 mm and 2.0 grams with a diameter of 0.8 mm) in the flask was placed in an oven for 2 hours at 65 °C and then carried the radiation of ultraviolet light for 2 hours in a microbiological chapel [15]. The substrate was moistened with sodium citrate buffer (0.1 M, pH 4.5) supplemented with ammonium sulphate 0.5% (w/v). The moisture content, pH, and temperature used are shown in Table 3. The fermentation was conducted for a period of 120 hours. The influence of temperature, pH and initial moisture on the response variables and the activities of phytase and xylanase were analysed according to the 2^3^ full factorial design [27] with four repetitions of the central point (Table 1). The choice of variables and their levels was made according [15,28]. All statistical analyses were performed using the software Statistica 8.0. 

**Table 3:** Full factorial design (2^3^) for the production of phytase and xylanase by solid state fermentation using citrus pulp as substrate.

Runs *	Variables		
	Moisture (%)	pH	Temperature (°C)
1	50	4.0	26
2	70	4.0	26
3	50	6.0	26
4	70	6.0	26
5	50	4.0	34
6	70	4.0	34
7	50	6.0	34
8	70	6.0	34
9 (C)	60	5.0	30
10 (C)	60	5.0	30
11 (C)	60	5.0	30
12 (C)	60	5.0	30

* The order of experiments was carried out randomly. (C) Central points.

### 3.4. Extraction of Enzymes

Enzyme extraction was performed every 24 hours. A portion (5 g) of the fermented mixture was mixed with 30 mL of 0.1 M citrate buffer (pH 4.5). After maceration, the extract was clarified by filtration and centrifugation at 5,000 × g for 15 min [15]. The clear extracts were used in a suitable dilution for phytase and xylanase activity determination.

### 3.5. Phytase Activity

Phytase activity was determined by quantification of the phosphate released from phytate during the enzymatic reaction using the method of ammonium molybdate with modifications described by Spier *et al.* [28]. A volume of sodium acetate buffer (350 μL, 100 mM, pH 4.5) containing sodium phytate (875 nmol) was used as substrate. After pre-incubation at 37 °C for 10 minutes, the enzymatic reaction was initiated by the addition of enzyme extract (50 μL). This homogenized solution was incubated for 30 minutes at 37 °C. Thereafter, AAM reagent (1.5 mL) containing ammonium molybdate 10 mM/H_2_SO_4_ 5N/acetone (1:1:2 v/v) and 1 M citric acid (100 mL) were then added. The release of the inorganic phosphorus was determined at an absorbance of 355 nm. One unit of phytase is defined as the amount of enzyme that releases 1 nmol of inorganic phosphorus per minute under the test condition. Enzyme activity was expressed in units per gram of dry basis (U/g). The standard curve was made with dibasic potassium phosphate with 10 to 600 nM phosphate per mL. All experiments were performed in triplicate.

### 3.6. Xylanase Activity

Xylanase activity was determined by mixing an appropriately diluted enzyme sample (0.1 mL) with birchwood xylan [Sigma, 1.0% (w/v)] in 50 mM sodium citrate buffer (pH 6.0, 0.9 mL) as substrate at 50 °C for 5 minutes [29]. Three milliliters of 3,5-dinitrosalicylic acid reagent was added to stop the reaction. The amount of reducing sugar released (as xylose) was estimated spectrophotometrically by measuring at wavelength of 540 nm [30]. One unit of xylanase was defined as the amount of enzyme required to release 1 μmol of reducing sugars per minute and the enzyme activity was expressed in units per gram of dry basis (U/g). All experiments were performed in triplicate.

## 4. Conclusions

Our results demonstrated the potential of solid state fermentation for the production of phytase and xylanase by *Lichtheimia blakesleeana*. It can use citrus pulp as substrate to produce hydrolytic enzymes that can increase the digestibility of animal feed ingredients, and can therefore be directly used as a dietary supplement for animals. The alternative approach for simultaneous production of phytases and xylanases is easy, simple and economic. It holds great potential for future biotechnological and industrial applications.
